# A systematic literature review of protective factors mitigating intimate partner violence exposure on early childhood health outcomes

**DOI:** 10.1111/jan.15638

**Published:** 2023-03-20

**Authors:** Kathryn J. Spearman, Emily Hoppe, Emma Jagasia

**Affiliations:** Johns Hopkins School of Nursing, Baltimore, Maryland, USA

**Keywords:** early childhood, intimate partner violence, positive childhood experiences, protective factors, resilience

## Abstract

**Aim:**

The objective of this integrative review was to critically synthesize the evidence on protective factors in early childhood that buffer the effects of exposure to intimate partner violence (IPV) on young children’s health outcomes.

**Methods:**

Studies were eligible for inclusion in this review if the article was (a) in English, (b) title or abstract discussed protective factors, buffering, resilience or mitigating factors in early childhood for young children who experienced IPV.

**Results:**

A total of 23 articles of 492 manuscripts identified from the search from peer-reviewed journals from 2010 to 2022 were included. Individual-level protective factors for young children exposed to IPV and include emotional self-regulation, child temperament and child self-esteem. Family-level protective factors were maternal physical and mental health; warm, responsive parenting; knowledge of child development; socioeconomic advantage; caregiver employment; and maternal education.

**Conclusion:**

The results of this integrative review highlight the critical importance of a dyadic approach to early childhood intervention. Health and legal systems should not only focus solely on pathology of family violence but also conceptualize treatment and courses of action from a strength-based perspective in order to empower victims of IPV, and promote the safety, health and well-being of children. Future research should examine the role of system-level protective factors.

**Impact:**

This review adds to the growing body of the evidence of positive relational health as a key social determinant of health for children. This will be foundational to design interventions that shield children from further harm and promote health, flourishing and recovery from violence and trauma.

## 1 | INTRODUCTION

Early caregiving experiences and family relational health are the central social context in which children live, learn, develop and play. These relational experiences are a profound social determinant of health for children. Social determinants of health affect a wide range of health outcomes and can be grouped into five domains, including economic stability, education access and quality, healthcare access and quality, neighbourhood and built environment, and the social and community context in which a person lives (Healthy People, 2030). Intimate partner violence (IPV) is a relational health risk that is associated with adverse health consequences in childhood and throughout the lifespan. Intimate partner violence involves willful intimidation of a current or former intimate partner and is characterized by patterns of abusive behaviours such as physical violence, sexual violence, coercion, threats, stalking, psychological or emotional aggression, and economic abuse (Breiding et al., 2015; Stark, 2007). The impact of IPV extends beyond the adult victim and has negative consequences for the entire family system (Howell et al., 2016).

Childhood exposure to IPV is a common experience: approximately 15.5 million children in the United States live in households with IPV, and up to 25% of children are exposed to IPV during their childhood (Finkelhor et al., 2015; McDonald et al., 2006). In addition to the impact of IPV on the social context, substantial research has demonstrated how IPV impacts economic stability and housing stability (Bethell et al., 2019; Daoud et al., 2016; O’Connor & Nepomnyaschy, 2020). Intimate partner violence exposure also impacts children’s education, with children exposed to IPV more likely to have learning disabilities and less likely to be engaged in school (Holmes et al., 2022; Ravi & Black, 2022). Intimate partner violence exposure in childhood also impacts healthcare access and quality: children exposed to IPV are more likely to have unmet health needs (King & Khanijahani, 2020).

Most studies relating to the risk and protective factors for childhood exposure to IPV and child maltreatment have focussed on school-aged and adolescent children and long-term outcomes in adults, despite the fact that children under five are more likely to be exposed to IPV (Dubowitz et al., 2016). Few studies have examined the impact of IPV exposure on young children and subsequent developmental trajectories (Bowen, 2017; Howell, 2011). In addition, while there is a growing body of literature that looks at the long-term impact of positive and adverse childhood experiences (PACEs) on adult health outcomes, there is less research that has looked at PACEs, protective factors and health outcomes in early childhood. Young children face an even greater vulnerability to threat given the sensitivity of their developing brains and hormonal systems (Machlin et al., 2019). Therefore, identifying protective factors in early childhood is crucial to developing interventions and policies that support child development.

Much of the existing literature on protective factors and positive childhood experiences that mitigate the impact of family violence on children focusses on factors external to the family, such as peer and social support. Young children are less likely to be buffered by factors external to the family given their dependence on caregivers and their inability to escape violence through peer or school outlets (Fogarty et al., 2020; Howell, 2011). Young children rely on parents and primary caregivers for both basic needs such as safety, as well as modelling for emotional regulation, a more advanced developmental task (Howell et al., 2016). Identifying protective factors in early childhood and designing interventions at each of the society, community, family and individual levels is crucial to shield children from further harm and to cultivate flourishing, health and recovery from violence and trauma (Schultz et al., 2013). A growing body of research suggests that positive relational experiences may outweigh trauma and adversity (Bethell et al., 2019; Grych et al., 2015). These positive relational experiences provide a buffer that can lessen the harmful impact of trauma exposure, by helping the young child re-establish a sense of safety. Thus, in order to help children overcome adversity, early childhood investments that promote positive relational development can create lasting benefits through the life course (Anderson Moore et al., 2017).

For these reasons, the purpose of this integrative review was to examine the literature over the past decade (2010–2021) to critically synthesize the evidence on protective factors in early childhood that buffer the effects of IPV on young children’s health outcomes. We defined early childhood as the years prior to school age, encompassing infancy, toddler and preschool-aged children ages 0–6 years old.

## 2 | BACKGROUND

### 2.1 | Resilience in early childhood: A concept defined

Protective factors, or buffers, are those attributes at each of the societal, community, family and individual levels that promote positive adaptation, mitigate adversity and predict better outcomes (Howell, 2011; Schultz et al., 2013). Resilience is a dynamic process involving an individual’s capacity to adapt, recover and rebound from adversity, and results from complex interactions with societal, community, family, individual, physiological and cellular factors across the life course (Schultz et al., 2013; Szanton & Gill, 2010). For young children exposed to IPV, resilience has been conceptualized as the ability to function successfully and achieve developmental goals following prolonged trauma exposures (Howell et al., 2010). Dubowitz et al. (2016) and Sattler and Font (2018) both conceptualized resilience as ‘competency’. Competency can be considered positive functioning and meeting the normative expectations of society in social, behavioural, and developmental domains (Dubowitz et al., 2016; Sattler & Font, 2018).

Yet, the absence of negative health does not mean that a child is flourishing. Flourishing can be defined as holistic positive development across all five domains of physical health and functioning, mental and emotional well-being, social and academic development, and relationships (Moore et al., 2017). The National Survey of Children’s Health (NSCH) measures flourishing for young children under five as how often the child is able to bounce back quickly when things do not go their way, how often a child is tender or affectionate, how often a child shows interest and curiosity in learning new things and how often does a child smile and laugh (Child and Adolescent Health Measurement Initiative (CAHMI), 2022). A flourishing child is a resilient child.

### 2.2 | Health consequences of early childhood exposure to adversity

Children bear a significant burden of morbidity and mortality related to IPV and maltreatment with lasting physical, mental and behavioural consequences. Consequences of children’s exposure to IPV, and health outcomes ranging from increased rates of asthma, hospitalizations, underimmunization, depression, anxiety, PTSD, speech or language disorders, learning disabilities, academic functioning and many other special health and developmental needs (Bair-Merritt et al., 2015; Felitti et al., 1998; Holmes et al., 2022). Health outcomes for children exposed to IPV and child maltreatment vary by age, developmental stage, chronicity and violence severity. Children are particularly vulnerable to exposure during sensitive developmental stages. For very young children, existing scholarship suggests that there are also ‘sleeper effects’ of IPV exposure; that is, physical and mental health sequalae that emerge years after exposure to IPV (Dubowitz et al., 2016). Through mechanisms that include stress-induced inflammation, immune dysregulation and disrupted neurodevelopment, relational health risks such as IPV, child maltreatment and other ACEs are linked in a dose–response relationship to many of the leading causes of morbidity and mortality in adulthood as well as future violence victimization and perpetration (Felitti et al., 1998; Franklin & Kercher, 2012). For children exposed to IPV and maltreatment, disrupted neurodevelopment occurs throughout childhood and into adulthood. During infancy and early childhood, exposure to IPV and/or maltreatment is associated with attenuated growth of structural areas of the brain relating to memory, emotional regulation, and auditory and visual processing exhibiting (Bethell et al., 2019).

Developmental risk and resilience may also transmit intergenerationally, with research studies linking parents’ childhood exposure to adversity to deleterious behavioural and health outcomes in children (Greene et al., 2018; Hendrix et al., 2020; Schickedanz et al., 2018). Although potential mechanisms for this transmission, including parenting practices, parental mental health, epigenetic changes, and perinatal exposures, are under study, intergenerational transmission of risk and resilience remains only partially understood (Narayan et al., 2021). Evidence suggests that at least some of this transmission occurs at the cellular level. For example, one study found that telomere length, a marker of cellular aging, was shorter in offspring of mothers who reported higher ACE exposure (Esteves et al., 2020). Research has examined epigenetic changes related to the cumulative stress of historical trauma, which is associated with pervasive health inequities in communities with historically oppressed identities (Brockie et al., 2013; Conching & Thayer, 2019; Heberle et al., 2020; Lehrner & Yehuda, 2018).

Not all young children who are exposed to IPV and child maltreatment suffer measurable adverse consequences. Complex, heterogeneous pathways of genes and environment impact development in young children exposed to family violence (Boyce et al., 2021). Minor changes in these factors can result in developmental trajectories of vulnerability or resilience that are dynamic and may change over time (Brockie et al., 2013). Early-life adversity is best viewed as influencing health, rather than as determining health. A deterministic approach obscures the complexities of environments and exposures that happen to an individual throughout a lifetime, including protective factors and exposures during other sensitive periods (Wallack & Thornburg, 2016). More is known about the pathology resulting from exposure to abuse than the protective factors that help children cope with traumatic exposure (Howell, 2011).

A recent large, US national study (*n* = 131,744) using data from the National Survey of Children’s Health (NSCH), found that children who faced relational health risks only (such as exposure to IPV or parental substance use) were more likely to have mental, emotional or behavioural conditions than children who faced social health risks only such as family economic hardship (Bethell et al., 2022). This supports the importance of examining IPV and relational health risks as crucial social determinants of health for children. However, warm, nurturing caregiving from nonoffending mothers may buffer stress responses in children and mediate the effects of exposure to violence (Berman et al., 2011). Positive relational health including positive childhood experiences (PCEs) related to secure attachment—what the CDC terms: ‘safe, supportive, and nurturing relationships’—have been shown to reduce the burden of illness related to childhood adversity (Bethell et al., 2019; CDC, 2019). These positive relationships are one of the most powerful forces of healing and resiliency in a child’s life.

## 3 | THE REVIEW

### 3.1 | Aim

The aim of this integrative review was to critically synthesize the literature on protective factors in early childhood (defined as including infancy, toddlers, preschool-aged children and children under 6) that mitigate the impact of childhood exposure to IPV, on young children’s physical, mental and behavioural health outcomes.

### 3.2 | Design

An integrative review method was used to critically examine sources including qualitative, quantitative, mixed methods and systematic reviews in order to provide a comprehensive overview. This process included identifying the problem and aim of the review, conducting a comprehensive search of the literature, synthesizing the evidence and presenting the findings (Whittemore & Knafl, 2005).

### 3.3 | Search methods

Databases including PubMed, Embase and CINAHL were searched with the following combinations of terms: ‘Intimate Partner Violence’[MeSH Terms] OR ‘Domestic Violence’[MeSH Terms] OR ‘Battered Women’[MeSH Terms] AND ‘childhood exposure’ OR ‘children’s exposure’[All Fields] OR ‘children’ OR ‘infant’ OR ‘pediatric’ AND ‘protective factor’ OR ‘positive childhood experiences’ OR ‘resilience’. Articles published over the last decade (2010–2022) were examined to capture the most recent literature on protective factors in early childhood.

### 3.4 | Search outcome

In the literature search, an article was eligible for inclusion in this review if it was (a) in English, (b) the title or abstract discussed protective factors, buffering, resilience or mitigating factors for children who had experienced or been exposed to family violence, specifically IPV and/or child maltreatment, (c) specifically discussed protective factors relating to early childhood, including infancy, toddlers, preschoolers, young children and (d) were limited to those published from 2010 through September 2022. Exclusion criteria included articles that were in reference to (a) community or neighbourhood violence or violence perpetrated by a nonfamily member (e.g. bullying, war trauma, youth serving organization, teacher and priest), (b) elder abuse or sibling abuse, (c) risk or protective factors for perpetrating violence, (d) protective factors related to adult victims of IPV, (e) articles that focussed on protective factors for primary prevention efforts (e.g. broad child sexual abuse prevention efforts in schools), (f) focus of study was on adult survivors of childhood IPV exposure or (g) systematic review articles that were based on articles published prior to 2010.

**FIGURE 1:**
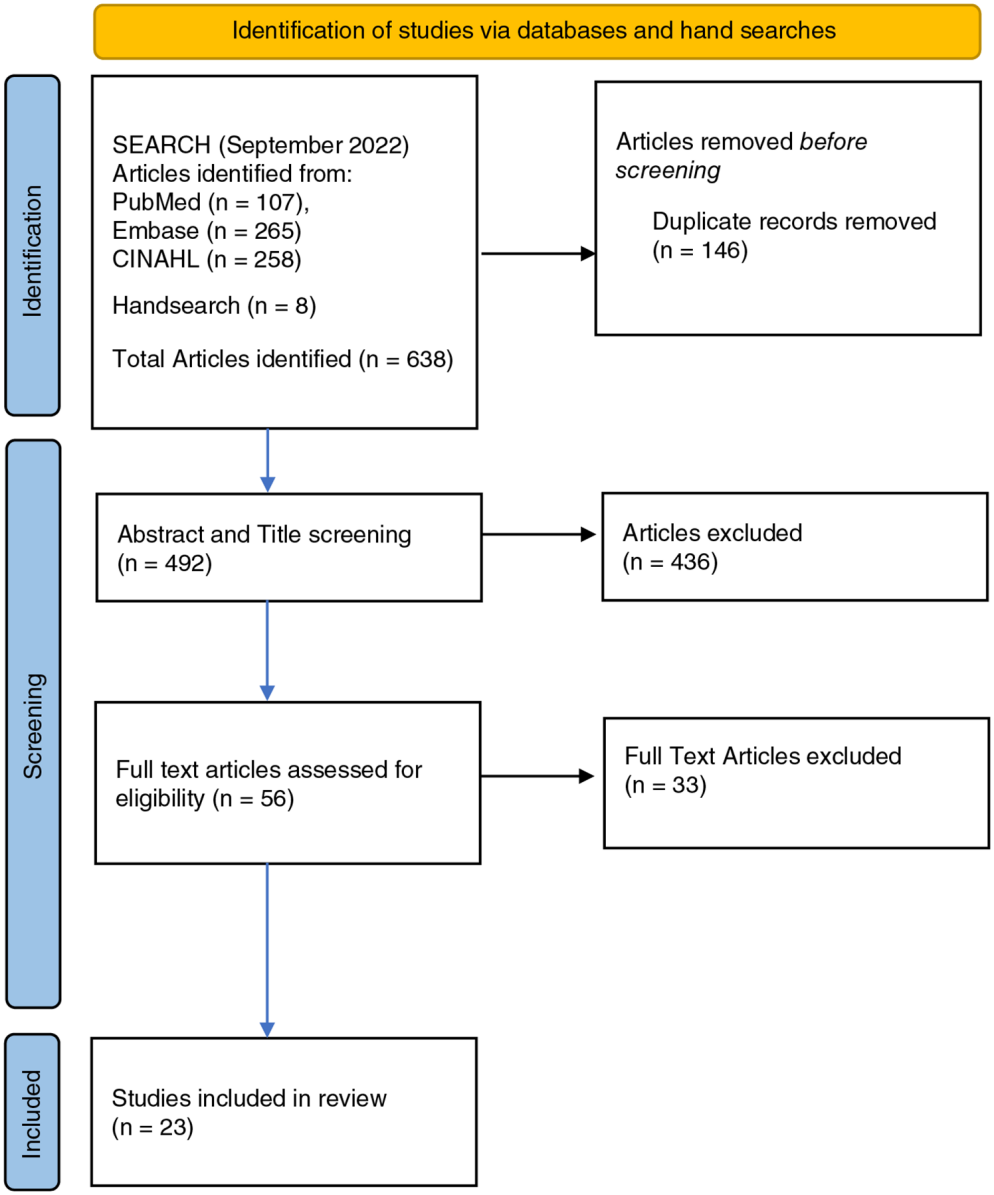
PRISMA diagram.

A total of 630 articles were retrieved by the search strategy and imported into Covidence, a systematic review software program that allowed the team to collaborate on the review. Eight additional articles resulted from review of references and a search on Google Scholar. One hundred and forty-six duplicates were removed, and 492 articles were screened for eligibility by the first author and reviewed by the second and third authors. Four hundred and thirty-six studies did not meet inclusion criteria during title and abstract screening. Fifty-six articles required full-text screening, and of those an additional 33 articles were excluded, leaving 23 articles meeting study inclusion and exclusion criteria (Figure 1: PRISMA diagram).

### 3.5 | Quality appraisal

During the extraction process, quality and level of evidence was assessed (Table 1) based on the Johns Hopkins Evidence-based Practice Toolkit (Dang & Dearholt, 2018). This tool organizes articles based on quality and level of evidence based on study design. Level I includes experimental studies or randomized control trials, Level II includes quasi-experimental studies including systematic reviews and Level III includes nonexperimental studies. Quality was appraised as high quality (A), good quality (B) or low quality (C) that denotes inconsistent results or insufficient sample size.

### 3.6 | Data abstraction and synthesis

Data were extracted by three independent researchers. The first author developed a predesigned template prior to extraction. Data from each article were synthesized across multiple domains using this template: individual factors, family and parenting factors, demographic factors, and violence factors as well as across the associated mental, behavioural and physical health outcomes (Table 1). The second and third author reviewed and appraised the articles separately. Any discrepancies between the second and third author were reviewed by the first author. Relevant information from each article selected was extracted into a summary table (Table 1) and guided by Bronfenbrenner’s (1994) ecological systems theory.

## 4 | RESULTS

This integrative review yielded 23 articles across various quantitative study designs and literature reviews that addressed protective factors in early childhood, with results and quality of evidence presented in Table 1. Outcome variables assessed in these studies included physical health outcomes, behavioural and mental health outcomes. The two studies (Hibel et al., 2011; Mueller & Tronick, 2019) identified in this integrative review that examined physical health outcomes focussed on adrenocortical dysregulation. Behavioural and mental health outcomes examined in these studies included conduct disorder, internalizing and externalizing symptoms, language disorders and traumatic stress or PTSD symptoms. For example, Fogarty et al. (2020) looked at mothers who had experienced partner violence at 12-month postpartum (15% of women reported IPV in first 12 months), and the children’s resilience for emotional/behavioural outcomes at 4 years of age. Resilient children were often defined as exposed to IPV but with lower than clinical levels of conduct disorder symptoms.

### 4.1 | Individual-level protective factors

Individual-level protective factors for young children exposed to family violence include intrinsic personality characteristics or traits. The pathways and mechanisms of how these individual level protective factors may mitigate exposure to IPV are not entirely elucidated in the literature, and the factors identified varied based on how they are measured. For example, in some studies, emotional regulation at the individual level was conceptualized as an individual trait that was protective (Sattler & Font, 2018), and in other studies, emotional regulation or self-control was an outcome as a result of the interaction between a child’s risk and protective factors (Schultz et al., 2013). However, despite these nuances in identifying individual protective factors, common themes across the studies included in this literature review indicated that emotional self-regulation, child temperament and child self-esteem were protective for children exposed to IPV (Bethell et al., 2019; Sattler & Font, 2018).

### 4.2 | Family-level protective factors

Family-level protective factors include parenting factors such as maternal and caregiver health; parenting practices, knowledge and responsiveness; demographic factors such as education and employment; and violence factors such as the cessation or reduction of violence in the family.

#### 4.2.1 | Parenting protective factors

Protective factors for young children exposed to family violence identified at the family level included maternal health and nurturing, sensitive parenting. Maternal health was identified in a number of studies as protective. Fogarty et al. (2020) further emphasized the importance of a healthy maternal caregiver, and other researchers hypothesized that mothers who experience good mental health may help children learn emotion regulation (Bethell et al., 2019; Howell et al., 2010). Dubowitz et al. (2016) identified that having a healthy parent who is not depressed is important to fostering resilience. In addition to maternal mental health, maternal physical well-being was identified as a significant predictor of resilience for young children, hypothesizing that mothers who have a higher degree of physical well-being may have higher energy levels and more able to provide a consistent, nurturing home environment (Bethell et al., 2019).

Parenting characterized by warmth, nurturing, responsiveness and ‘appropriate discipline’ is protective and helps facilitate development in emotional regulation and prosocial skills (Howell et al., 2010). Maternal understanding of infant and child development (measured by the Knowledge of Infant Development) may moderate associations between maternal traumatic experiences and child socio-emotional behavioural problems (Ahmad, Rudd, et al., 2022; Ahmad, Shih, et al., 2022). Even after controlling for violence severity and demographic variables such as income, better parenting performance and maternal mental health were protective (Howell et al., 2010).

Higher levels of maternal sensitivity, supportiveness and scaffolding during mother–child interactions was identified as a protective factor in a longitudinal study by Ahmad, Rudd, et al., 2022; Ahmad, Shih, et al., 2022. Other researchers such as Hibel et al. (2011) also identified early maternal sensitivity as buffering the impact of exposure to violence in toddlers. Frequent activation of the body’s stress response system (hypothalamic pituitary adrenal (HPA) axis) leads to inappropriate amounts of adrenaline and cortisol in the young child and can lead to structural changes in the child’s brain (Jonson-Reid & Widerman, 2017), In a study by Hibel et al. (2011), dysregulation in cortisol levels first appeared in toddlers exposed to accumulated levels of violence at age of 24 months, but early maternal sensitivity appeared to buffer this impact.

One of the most frequently measured behavioural health outcomes were children’s internalizing and externalizing behaviours, including conduct disorders. The literature review conducted by Howell et al. (2016) cited several studies that have shown that about 35% of young children exposed to IPV score in clinical range for adjustment problems, about 20% demonstrate resilience, and the remainder score borderline. Howell et al. (2010) found that preschoolers exposed to IPV tend to have increased externalizing and internalizing behavioural problems, increased aggression, lower intellectual functioning and lower social functioning. Protective factors for a child’s PTSD symptoms included maternal satisfaction in parenting and other parenting factors. Maternal satisfaction reduced the IPV survivor-mother’s own PTSD symptoms, suggesting it is a protective factor for both mothers and children. Based on the DSM-5 criteria for PTSD in children under ages 6, it is possible that 50% of young children following exposure to IPV experience clinically significant symptoms of PTSD (Howell et al., 2016). Schultz et al. (2013) found that a child’s internal locus of self-control and more responsive parenting was associated with lower PTSD symptomatology, suggesting both individual and family factors that are protective in situations of family violence.

#### 4.2.2 | Demographic protective factors

Demographic factors identified in the studies as protective factors for young children exposed to IPV include maternal education, employment and socio-economic advantage. In this integrative review, demographic protective factors were mentioned for studies that focussed on behavioural outcomes, but not in studies that examined physical or mental health outcomes. Socio-economic advantage and maternal education were found to have a protective effect when measuring behavioural outcomes in young children (Bowen, 2017). Bowen (2017) found that socio-economic advantage buffers the impact of risk on development of conduct disorder symptoms, in addition to maternal sensitivity and positive parenting. Socio-economic advantage buffers the impact of risk on the development of conduct disorder symptoms. Higher maternal education more than doubled the likelihood of resilience for boys and increased the likelihood of resilience for girls (Bowen, 2017). Dubowitz et al. (2016) identified that caregiver employment was associated with resilience, but household income was not a significant predictor of resilience. Fogarty et al. (2020) identified maternal return to work or study as a significant predictor of children’s behavioural outcomes, consistent with other studies showing maternal education and employment as protective. Maternal employment was linked with leaving IPV relationships, reduced violence exposure for children and increased self-esteem in mothers and children.

#### 4.2.3 | Cessation or reduction in violence as protective factors

The level and severity of exposure of violence significantly impacts child outcomes (Howell, 2011). Violence factors include the developmental timing of exposure to violence and the severity of violence exposure. Holmes et al. (2017) found that IPV exposure during the toddler years significantly predicted childhood aggression during preschool years. The significant role of early intervention was highlighted by Fogarty et al. (2020): when exposure to IPV stops at an early age, children were more likely to demonstrate emotional-behavioural resilience. Fogarty et al. (2020) found that for children who had been exposed to IPV at 12-month postpartum but were no longer exposed to IPV between ages 3 and 4 demonstrated greater levels of emotional-behavioural resilience. This cessation of violence exposure indicated some type of protective shift in the family such as either the mother had left the IPV relationship or the partner sought support. Howell et al. (2010) found that violence against the mother was a good predictor of children’s exposure even when it was not directly assessed. Few studies explicitly identified cessation of violence as a protective factor that would allow a child to heal following exposure, suggesting this is an area for further research. Only two studies identified lower violence severity as a factor influencing resilience (Howell et al., 2010, 2013).

## 5 | DISCUSSION

The results of this integrative review illuminate opportunities to mitigate the effects of IPV on young children. As is supported by previous research with older children, a number of studies in this integrative review highlighted the importance of maternal factors including mental and physical health, as well as employment and education, as protective. The mother’s ability to cope with adversity resulting from IPV experiences in turn impacts the child’s ability to cope, thus influencing the child’s resilience (Howell, 2011). While women exposed to IPV have higher rates of depression and trauma symptoms than women not exposed, a mother’s capacity for providing her child with effective coping mechanisms and conflict resolution strategies—despite violence—helps positively affect the child’s competence in social and emotional domains (Howell et al., 2010).

The literature review on protective factors for children exposed to IPV has several strengths. Some of the included studies involved longitudinal cohort studies that identified exposed or high-risk children during early childhood and then followed their developmental trajectories into adolescence. These studies provide rich information on the multiple mediating and moderating influences on resilience in childhood, as well as the role of time. Additionally, many of the studies took a strength-based approach to understanding the risks and protective factors for young children exposed to IPV, which sheds light not only on pathological processes, but also on processes supporting children’s well-being. These strengths are particularly salient when considering potential clinical and policy responses to early childhood exposure to IPV.

### 5.1 | Implications for nursing

Healthcare systems should not only focus solely on pathology of family violence but also conceptualize treatment and courses of action from a strength-based perspective building on families’ strengths, empowering victims of IPV, and promoting safety, health and well-being of children (Howell et al., 2010). Nurses and advanced practice providers should assess families for relational health risks and strengths during each healthcare visit by asking about the state of the parents’ relationship, and the relationship between parent and child. Common questions supported by the American Academy of Pediatrics are seen in Table 2 and include questions pertaining to parents perceived safety and support as well as parent–child attachment and bonding (Hagan et al., 2017). Managing these social determinants of health is a key aspect of holistic, proactive patient care (Tiase et al., 2022). None of the articles identified as part this review were from nursing disciplines, suggesting the need for more nursing research into this field and transdisciplinary approaches with public health, law, family science, psychology to identify ways to support families and promote healthier environments for children. Numerous opportunities exist for nursing interventions at the individual and family level to promote the health and well-being of children following exposure to IPV (Table 3). This review highlights the importance of a two-generation, strength-based approach to promoting the health and well-being of children. Importantly, parents with young children should be supported in recovering from their own trauma to help ensure that their mental health symptoms and parenting is not adversely impacting their children’s emotional and physical health (Greene et al., 2018). In addition, improving access to mental health services and treatment for caregivers who have experienced violence could offer a protective effect for children. Nurses play an important role in assessing caregiver mental health and linking survivors and their children to needed resources. Nurses can also advocate for strength-based approaches to parenting, and/or refer families to evidence-based parenting and therapeutic programmes and home visiting programmes.

Family-level demographic factors such as maternal education and employment are protective factors and are influenced by other social determinants of health. While these demographic factors are at the family level, policies and system-level interventions that reduce barriers to mothers obtaining education and employment could be effective interventions at promoting resiliency (Table 3). Nurses play an important role in advocacy, both for their patients and by addressing factors at the systems level. Some examples of societal-level factors that could be protective are policies such as affordable or universal childcare, paid maternal leave and other policies that reduce barriers for mothers to obtain employment. In addition, given the importance of violence cessation as a protective factor, this suggests needed support for mothers and children in the aftermath of leaving an abusive partner/parent. In this example, the family court plays a central role in determining the child’s environment postseparation and preventing ongoing IPV postseparation (Spearman et al., 2022). Family court judges have wide discretion in crafting custody and access orders that promote safety and health for children. More collaboration is needed across sectors including law, public health and social services to address the health implications of judicial decisions, an important social determinant of health since judge’s decisions directly impact a child’s environment.

### 5.2 | Limitations

One of the primary limitations of the literature examined is the cross-sectional nature of how resilience is measured. Resilience can vary over time and across domains. Another limitation is that most of these studies rely on mothers as the only informant on the level of violence and on children’s behaviour. Intimate partner violence disproportionately affects women, mothers and children; however, the role of fathers, both as perpetrators or as victims of violence, was largely ignored in the literature. The studies included in this literature review also did also not specifically examine children’s exposure to violence in same-sex parental relationships. The research has failed to discuss other early exposures that may predispose young children to vulnerability or genetic variables that may influence trajectories. No studies explored societal-level protective factors such as policies, family court interaction, child removal or contact with child welfare agencies that have a substantial impact on family function. Finally, most of the articles included in this review addressed US or UK populations, such that a truly international perspective is lacking.

This study demonstrated multiple areas for further research. Understanding protective factors for paediatric physical health outcomes (e.g. somatic complaints and autoimmune disorders) is an area in need of additional exploration. The current literature demonstrates a lack of investigation into intersectional impacts of racism and discrimination with IPV exposure. While race is often included as a covariate in data analyses, the historical and structural factors leading to differences in health impact and protection by race are excluded from examination. Furthermore, while cessation of violence exposure or reduction in severity of violence exposure have been hypothesized as being protective, this has not been adequately explored in the literature. Little attention has been given to the factors that may lead to violence cessation, such as either a change in perpetrator behaviour or a separation from the abusive partner. However, survivors of IPV who wish to leave an abusive relationship must continue to navigate co-parenting relationships, and the structural context of family court (Spearman et al., 2022). Children are vulnerable to exposure to postseparation abuse, or ongoing IPV following parental separation/divorce, as they transition between households (Spearman et al., 2022).

## 6 | CONCLUSION

Despite these limitations, this integrative review highlights the importance of promoting warm, nurturing relationships and responsive parenting that helps children with emotional regulation in order to mitigate the harmful effects of IPV exposure on health outcomes in early childhood. Given the important role that maternal mental and physical health has on paediatric outcomes for children exposed to IPV, this literature review highlights the necessity of a two-generation, dyadic approach in mitigating early childhood exposure to IPV. Moreover, focussing on maternal mental and physical health and providing job and educational opportunities suggests a priority for future interventions and policy. Promoting positive relational health is a priority for all children, and especially for those who have experienced adversity. There is a need for a focus on the structural-level determinants of a child’s home environment, including judicial decisions on custody, laws and policies that impact parental education, employment and opportunities for all children to live safe, healthy lives.

## Figures and Tables

**TABLE 1:** Articles identified in systematic literature review of protective factors in early childhood mitigating IPV on young children’s health outcomes.

Author, year	Study design	Level and quality of evidence	Study location	Sample size	Age of children	Paediatric outcome domain	Individual factors			Family-level factors								
										Parenting				Demographic			Violence	
							Child temperment	Emotional self-regulation	Cognitive factors	Caregiver mental well-being	Maternal physical well being	Maternal parenting (sensitivity, attachment, interaction, satisfaction, routines)	Breast-feeding	Socio-economic advantage	Employed caregiver	Maternal education	Lower Violence severity	Cessation of IPV exposure
Behavioural outcomes																		
Ahmad, Rudd, et al. (2022) and Ahmad, Shih, et al. (2022)	Longitudinal cohort	Level II A	United States (Tennessee)	1127	Prenatal exposure, infant outcome	Child socioemotional and behavioural problems						✕						
Ahmad, Rudd, et al. (2022) and Ahmad, Shih, et al. (2022)	Longitudinal cohort	Level II A	United States (Tennessee)	1034	Ages 4–6	Executive functioning and externalizing problems						✕						
Bowen (2017)	Longitudinal cohort	Level II A	Britain	7743	4 years old	Conduct Disorder Symptoms		✕				✕		✕		✕		
Bowen (2015)	Longitudinal cohort	Level IIA	Britain	7712	4 years old	Child peer problems		✕		✕		✕						
Dubowitz et al. (2016)	Prospective cohort	Level II A	United States (East, Midwest, South, Southwest, Northwest)	943	4–6 years old	Competency in behavioural, social, and developmental domains				✕					✕			
Fogarty et al. (2020)	Longitudinal cohort	Level II A	Australia	1060	4 years old	Emotional and behavioural symptoms				✕	✕				✕			✕
Galano et al. (2022)	Longitudinal cohort	Level II A	Southeast Michigan and Ontario	120	Preschool (ages 4–6)	Child resilience, child behaviour problems, childhood irritability		✕		✕		✕						
Holmes et al. (2017)	Longitudinal cohort	Level II A	United States	1399	Toddler (age 2–3); Preschool (age 3–4)	Child aggression						✕				✕		
Holmes et al. (2018)	Longitudinal cohort	Level II A	United States	1776	0–5 years old	Language/academic functioning	✕					✕		✕		✕		
Howell et al. (2010)	Cross-sectional study	Level II C	United States (Michigan)	56	4–6 years old	Behavioural problems				✕		✕					✕	
Howell et al. (2012)	Cross-sectional study	Level II C	US (Southeast Michigan) and Canada (Ontario)	52	Preschool age	Child internalizing, externalizing behaviours, prosocial skills		✕										
Howell et al. (2013)	Randomized control trial	Level I B	United States (small Midwestern cities)	113	4–6 years old	Emotion regulation and prosocial skills				✕							✕	
Peterson et al. (2019)	Prospective longitudinal	Level II C	United States	79	Birth to 3 years old	Child language development						✕				✕		
Sattler & Font (2018)	Longitudinal cohort	Level II A	United States	1193	Under 12 months at wave 1	Social-emotional and cognitive domains	✕	✕	✕			✕						
Mental & cognitive health outcomes																		
Cohodes et al. (2016)	Cross-sectional study	Level II B	United States, San Francisco, CA	95	3–5 years old	Cognitive functioning, verbal capacity		✕				✕						
David et al. (2015)	Cross-sectional study	Level II B	US, southeast	83	3–6 years old	School readiness						✕					✕	
Greene et al. (2018)	Prospective cohort	Level II B	United States (Chicago)	308	3–6 years old	Child psychiatric symptoms				✕								
Howell (2011)	Literature review	Level III C	N/A	N/A	Preschool age	Psychopathology				✕		✕					✕	
Miller-Graff & Howell (2015)	Longitudinal cohort	Level II A	United States	1178	4 years old at exposure	Posttraumatic stress symptoms						✕					✕	
Miller-Graff & Scheid (2020)	Longitudinal cohort	Level II C	United States	82	4 months	Posttraumatic stress symptoms							✕					
Schultz et al. (2013)	Longitudinal case control	Level II B	United States	350	1–12 years	PTSD and behavioural symptoms		✕		✕		✕						
Physiologic and physical health outcomes																		
Hibel et al. (2011)	Longitudinal cohort	Level II A	United States, nonurban (North Carolina & Pennsylvania)	1102	7–24 months	Adrenocortical response to emotion challenge tasks						✕						
Mueller and Tronick (2019)	Literature review	Level III B	N/A	N/A	Infancy, early childhood	Cognitive development, HPA axis, and auditory and visual cortex						✕						

**TABLE 2:** Nursing assessment of relational health.

Parents’ relationship/presence of IPV	‘How does your relationship with your partner or co-parent make you feel?’
Parent-child relational health	‘What do you enjoy doing with your child?’ ‘What do you think are your child’s strengths?’

**TABLE 3:** Nursing interventions to promote well-being of young children exposed to IPV.

Individual-level factors	Assess and promote positive relational health and emotional well-being during all encounters Provide trauma-informed care as IPV is common, and patients may not disclose Educate parents on emotional regulation and provide tools and resources to teach and model emotional regulation to children. Health promotion resources include: https://www.zerotothree.org/ and therapeutic resources includes: https://www.erikson.edu/fussy-baby-network/
Family-level factors	Screen for IPV; for example, using tools for healthcare providers from Futures Without Violence (ipvhealth.org) Screen for IPV and child maltreatment Screen for caregiver mental health concerns Screen for child and family strengths to bolster relational health Educate parents on the role of safe, supportive and nurturing relationships to mitigate adversity Provide links to evidence-based, trauma-informed therapeutic interventions for children and families experiencing IPV Connect parents to evidence-based parenting and health-promotion programs such as home visiting programs Provide referrals to parent-focussed and dyadic therapeutic interventions for parental mental health Nurses can connect survivors and their children to domestic violence advocacy organizations, including those that offer legal aid, and provide wrap around services. Some states and jurisdictions may have address confidentiality programs and other services that can increase safety for survivors and reduce violence exposure for children. Connect parents with social services to promote food, housing and financial security
